# Circulating plasmablasts and follicular helper T-cell subsets are associated with antibody-positive autoimmune epilepsy

**DOI:** 10.3389/fimmu.2022.1048428

**Published:** 2022-12-08

**Authors:** Atsushi Hara, Norio Chihara, Ritsu Akatani, Ryusei Nishigori, Asato Tsuji, Hajime Yoshimura, Michi Kawamoto, Yoshihisa Otsuka, Yasufumi Kageyama, Takayuki Kondo, Frank Leypoldt, Klaus-Peter Wandinger, Riki Matsumoto

**Affiliations:** ^1^ Division of Neurology, Kobe University Graduate School of Medicine, Kobe, Japan; ^2^ Department of Neurology, Kyoto University Graduate School of Medicine, Kyoto, Japan; ^3^ Department of Neurology, Kobe City Medical Center General Hospital, Kobe, Japan; ^4^ Department of Neurology, Hyogo Prefectural Amagasaki General Medical Center, Amagasaki, Japan; ^5^ Department of Neurology, Kansai Medical University Medical Center, Moriguchi, Japan; ^6^ Neuroimmunology, Institute of Clinical Chemistry, University Hospital Schleswig-Holstein, Kiel, Germany; ^7^ Department of Neurology, University Hospital Schleswig-Holstein, Kiel, Germany

**Keywords:** autoimmune epilepsy, autoimmune encephalitis, plasmablasts, T follicular helper cells, ICOS

## Abstract

Autoimmune epilepsy (AE) is an inflammatory disease of the central nervous system with symptoms that have seizures that are refractory to antiepileptic drugs. Since the diagnosis of AE tends to rely on a limited number of anti-neuronal antibody tests, a more comprehensive analysis of the immune background could achieve better diagnostic accuracy. This study aimed to compare the characteristics of anti-neuronal antibody-positive autoimmune epilepsy (AE/Ab(+)) and antibody-negative suspected autoimmune epilepsy (AE/Ab(-)) groups. A total of 23 patients who met the diagnostic criteria for autoimmune encephalitis with seizures and 11 healthy controls (HC) were enrolled. All patients were comprehensively analyzed for anti-neuronal antibodies; 13 patients were identified in the AE/Ab(+) group and 10 in the AE/Ab(-) group. Differences in clinical characteristics, including laboratory and imaging findings, were evaluated between the groups. In addition, the immunophenotype of peripheral blood mononuclear cells (PBMCs) and CSF mononuclear cells, particularly B cells and circulating Tfh (cTfh) subsets, and multiplex assays of serum and CSF were analyzed using flow cytometry. Patients with AE/Ab(+) did not show any differences in clinical parameters compared to patients with AE/Ab(-). However, the frequency of plasmablasts within PBMCs and CSF in patients with AE/Ab(+) was higher than that in patients with AE/Ab(-) and HC, and the frequency of cTfh17 cells and inducible T-cell co-stimulator (ICOS) expressing cTfh17 cells within cTfh subsets was higher than that in patients with AE/Ab(-). Furthermore, the frequency of ICOS^high^cTfh17 cells was positively correlated with that of the unswitched memory B cells. We also found that IL-12, IL-23, IL-6, IL-17A, and IFN-γ levels were elevated in the serum and IL-17A and IL-6 levels were elevated in the CSF of patients with AE/Ab(+). Our findings indicate that patients with AE/Ab(+) showed increased differentiation of B cells and cTfh subsets associated with antibody production. The elevated frequency of plasmablasts and ICOS expressing cTfh17 shift in PBMCs may be indicative of the presence of antibodies in patients with AE.

## Introduction

Epilepsy is a chronic debilitating disease affecting 0.5–1.0% of the world’s population (1). The etiology of epilepsy varies and remains unknown. It has been reported that some cases in which seizures are refractory to antiepileptic drugs can be suppressed by immunotherapy (2). In these cases of patients with autoimmune epilepsy (AE), autoantibodies targeting neuronal surface and intracellular antigens, also called as anti-neuronal antibodies, have been identified. Antibodies against neuronal surface antigens are not only diagnostic markers but also pathogenic factors (3). The incidence of AE is estimated to be approximately 5–7% in adults with epilepsy (4). Diagnosis and treatment tend to be delayed because diagnostic antibody tests depend on a cell-based assay (CBA) that involves a highly sensitive and specific antibody analysis with limited availability.

Recently, APE2 and ACES scores have been reported as diagnostic criteria for autoimmune epilepsy as a predictor of anti-neuronal autoantibodies (5, 6). However, more inclusive criteria for autoimmune encephalitis proposed by Graus et al. in 2016 are widely used to avoid misdiagnosis of treatable autoimmune epilepsy (7). Some cases of epilepsy refractory to antiepileptic drug that meet Graus’s criteria for possible autoimmune encephalitis are negative for anti-neuronal antibodies, and even include noninflammatory temporal lobe epilepsy with newly developed seizures or epilepsy that is often difficult to distinguish (8–11). This could lead to a false negative result due to the timing of analysis, other unknown anti-neuronal antibodies, or noninflammatory etiology. In clinical practice, some patients who are negative for anti-neuronal antibody respond to immunotherapy, but the psychological side effects of corticosteroid may make it difficult to accurately determine the effects of treatment (9, 10). Nevertheless, aggressive immunotherapy is necessary, especially when anti-neuronal antibodies against cell surface antigens are positive (11). To decrease the discrepancy between bedside and bench, new biomarkers are required to distinguish the presence of anti-neuronal antibodies in cases of suspected autoimmune epilepsy. However, little is known about the immunopathological background of anti-neuronal antibody-positive autoimmune epilepsy during the active period. It has been reported that anti-neuronal antibodies and other autoantibodies are produced by plasmablasts and long-lived plasma cells with the support of T follicular helper cells (Tfh) (12) Tfh are defined as CD4^+^ T cells that express C-X-C motif chemokine receptor 5 (CXCR5) in secondary lymphoid tissues (13), and there is considerable clonal overlap between Tfh from lymphoid tissues and circulating T follicular helper cells (cTfh) from peripheral blood mononuclear cells (PBMCs) (14). Generally, cTfh cells are classified into three subsets: cTfh1 (C-X-C motif chemokine receptor 3(CXCR3)+ and C-C chemokine receptor 6(CCR6)−, cTfh2 (CXCR3−CCR6−), and cTfh17 (CXCR3−CCR6+). The cTfh2 and cTfh17 classes switch naive B cells to promote IgG production (15). In several antibody-associated autoimmune diseases, cTfh shift to cTfh17 or cTfh2 (16–20) and increase the cTfh17/cTfh1 cells ratio (21). Notably, the expression of inducible T-cell co-stimulator (ICOS) in cTfh and subset changes in cTfh have been identified as factors that promote B cell differentiation and antibody production (15, 22, 23). Here, we performed comprehensive antibody and lymphocyte subset analyses of B cells and cTfh in 23 patients with suspected autoimmune epilepsy who met Graus’s criteria for possible autoimmune encephalitis. The results were compared with clinical evaluations, and diagnostic biomarkers in the active phase of antibody-positive autoimmune epilepsy were discussed.

## Materials and methods

### Patients

This prospective multicenter study was conducted in Japan between January 2016 and May 2022. We recruited patients who visited the Division of Neurology, Kobe University Graduate School of Medicine, Kobe, Japan; Department of Neurology, Kobe City Medical Center and, General Hospital, Kobe, Japan; Department of Neurology, Hyogo Prefectural Amagasaki General Medical Center, Amagasaki City, Japan. Patients who met the following criteria were included in the study:1) had seizures and adequate clinical evaluation to differentiate autoimmune epilepsy, 2) met Graus’s criteria for possible autoimmune encephalitis (7), 3) had been comprehensively analyzed for known anti-neuronal antibodies in cerebrospinal fluid (CSF) or serum, and 4) did not receive oral steroid therapy or immunosuppressive therapy. We recruited age- and sex-matched healthy controls (HC). Patients with active phase epilepsy underwent brain MRI and electroencephalography (EEG), and their peripheral blood and CSF samples were collected before initiating intravenous methylprednisolone therapy. The exclusion criterion was previous intravenous corticosteroid treatment within three months before sampling. MRI abnormalities were defined as hyperintense signals on T2WI or fluid-attenuated inversion recovery in multiple regions, including the medial temporal lobe, gray matter, white matter, or all, reflecting inflammation. EEG abnormalities were defined as focal epileptic or slow-wave activities.

### Anti-neuronal antibody analysis

Anti-neuronal antibodies were analyzed as follows. CSF was analyzed using a CBA for the N-methyl-D-aspartate receptor (NMDAR) antibody (Euroimmun AG, Lübeck, Germany) and leucine-rich glioma-inactivated protein 1 (LGI1) antibody (Cosmic Corporation, Tokyo, Japan). Serum samples were analyzed using a CBA for myelin oligodendrocyte glycoprotein (MOG) antibody (Cosmic Corporation, Tokyo, Japan) and ELISA for GAD antibody (SRL, Tokyo, Japan). If the results were negative, we performed a screen immunohistochemistry using rat brain, as previously reported (24, 25). In brief, fresh rat brains were fixed by 4% paraformaldehyde for 30 min, following dehydrated in 40% sucrose in PBS and keep in 4°C overnight. Then, brains were frozen in liquid nitrogen, and sliced into 7 µm sections on a cryostat, and transferred onto coverslips. The brain slice on coverslips were treated with 0.3% H2O2 in PBS after wash, and blocked with 5% goat serum, followed by incubation with patients’ serum at 1:200 or CSF at 1:4 in blocking solution overnight in 4°C. The coverslips were washed and incubated with goat anti-human IgG (H+L) biotinylated antibody (#BA-3000, Vector, CA, USA), followed by staining with the ABC Elite Kit (#PK6100, Vector, CA, USA). The sections were analyzed by at least two conditions blinded experienced investigators using a Zeiss Axioscope (Zeiss, CA, USA).

Samples positive for rat brain screen with immunohistochemistry were further investigated for reactivity against specific neuronal antigens including α-amino-3-hydroxy-5-methyl-4-isoxazole propionic acid receptor (AMPAR), γ-aminobutyric acid type B receptor (GABA_B_R), contactin-associated protein-like 2 (CASPR2), Delta/Notch-like epidermal growth factor-related receptor (DNER), Zic4, dipeptidyl-peptidase-like protein 6 (DPPX), collapsin response mediator protein 5 (CRMP-5/CV2), Hu, Yo, Ri, Ma, and amphiphysin. To this end, we used standardized commercially available test kits [immunofluorescence tests with tissue, fixed transfected cells, enzyme-linked immunosorbent assay, and immunoblotting appropriately (Euroimmun, Lubeck, Germany)]. We defined patients with antibodies to these known antigens as antibody-positive autoimmune epilepsy: AE/Ab(+), and those with antibodies negative for rat brain immunohistochemistry but who met the inclusion criteria: AE/Ab(-).

### Flow cytometry

PBMCs were separated by density centrifugation using a Ficoll-Paque PLUS (GE Healthcare, Uppsala, Sweden). PBMCs were stained for B cell and cTfh subsets, and CSF samples were stained for B cell subsets using antibodies (**Supplementary Table 1**). Data were obtained using a FACSVerse or LSRFortessa X-20 (BD Biosciences, Franklin Lakes, NJ, USA). Negative expression was defined as the fluorescence minus one control. All fresh samples were analyzed within 24 h of collection.

Circulating B-cell subsets (plasmablasts, CD19^int^CD27^high^CD38^high^CD180− B cells; naïve B cells, CD19+CD27−IgD+ B cells; unswitched memory B cells, USM, CD19+CD27+IgD+ B cells; switched memory B cells, SWM, CD19+CD27+IgD- B cells; double negative B cells, DN, CD19+CD27−IgD− B cells) circulating Tfh subsets (circulating follicular helper T cells, cTfh, CD3+CD4+CXCR5+ T cells; cTfh1, CXCR3+CCR6−CXCR5+CD4 T cells; cTfh2, CXCR3−CCR6−CXCR5+CD4 T cells; cTfh17, CXCR3−CCR6+CXCR5+CD4 T cells), and their expression rate of ICOS (ICOS^high^cTfh, CD3+CD4+CXCR5+ICOS^high^CD4 T cells; ICOS^high^cTfh1, CXCR3+CCR6−CXCR5+ICOS^high^CD4 T cells; ICOS^high^cTfh2, CXCR3−CCR6−CXCR5+ICOS^high^CD4 T cells; ICOS^high^cTfh17, CXCR3−CCR6+CXCR5+ICOS^high^CD4 T cells) were analyzed. The phenotypes of B cell and cTfh subsets are shown in **Supplementary Table 2**.

### Serum and CSF cytokine and chemokine levels

Serum and CSF samples were collected during the active phase before intravenous methylprednisolone administration. Both serum and CSF were frozen at −80°C. Cytokine/chemokine production was measured using the LEGENDplex Human Inflammation Panel 1 (BioLegend, San Diego, CA) and BD™ CBA Flex Set System (BD Bioscience) using an LSRFortessa X-20 cell analyzer (BD Biosciences) and BD™ Human Soluble Protein Master Buffer Kit (BD Bioscience) according to the manufacturer’s instructions. IFN-γ, IL-6, IL-12p70, IL-17A, and IL-23 levels were analyzed using the LEGENDplex Human Inflammation Panel 1 and IL-4 levels were analyzed using the BD™ CBA Flex Set System.

### Data analysis

Flow cytometry data were analyzed using FlowJo software ver. 10.8.1 (BD Biosciences). Statistical analyses were performed using GraphPad Prism 9 software (GraphPad Software, San Diego, CA, USA). In addition, the two-sided unpaired *t*-test, two-sided Mann-Whitney *U* test, and Spearman’s correlation test were used as appropriate.

### Standard protocol approval, registration, and patient consent

This study was approved by the ethics committee of Kobe University Hospital (Nos.1381 and B190152), and signed informed consent was obtained from all participants.

## Results

### Patient characteristics

We included 23 patients with suspected autoimmune epilepsy and 11 HC. The patient subgroups are summarized in **Table 1**. Among the 23 patients, 13 (57%) showed the presence and 10 (43%) showed the absence of anti-neuronal antibodies, categorized as the anti-neuronal antibody-positive autoimmune epilepsy (AE/Ab(+)) group and antibody-negative suspected autoimmune epilepsy (AE/Ab(-)) group, respectively. No samples tested positive in rat brains and negative in a CBA. The mean age was 43.8 ± 18.0, 50.2 ± 19.2, and 34.1 ± 6.7 (mean ± SD) for patients with AE/Ab(+), AE/Ab(-), and HC, respectively. Five patients with AE/Ab(+) (38%) were positive for anti-neuronal antibodies against the N-methyl-D-aspartate (NMDA) receptor, four patients (31%) had antibodies against myelin oligodendrocyte glycoprotein (MOG), and four patients (31%) against leucine-rich glioma-inactivated 1 (LGI1). All patients with MOG antibodies had unilateral cortical FLAIR-hyperintense lesions in MOG-associated encephalitis with seizures subtypes of MOG-associated disease (26). There were no differences or bilateral abnormalities on MRI, several CSF abnormalities (cell counts, levels of protein, IgG index, oligoclonal bands), EEG, or tumor comorbidity between the AE/Ab(+) and AE/Ab(-) groups.

**Table 1:** Characteristics of patients with autoimmune epilepsy and HC.

	Antibody-positive autoimmune epilepsy (n=13)	Antibody-negative suspected autoimmune epilepsy* (n=10)	HC (n=11)	Antibody-positive autoimmune epilepsy vs. antibody-negative suspected autoimmune epilepsy, p value	Antibody-positive autoimmune epilepsy vs. HC, p value
Age(y), mean ± SD	43.8±18.0	50.2±19.2	34.1±6.7	0.44	0.15
Sex, female, n (%)	5 (38)	3 (30)	4 (36)	0.97	0.78
Anti neuronal-antibody, n (%)	NMDA 5 (38) MOG 4 (31) LGI1 4 (31)	–	–	–	–
Abnormal MRI findings, n(%)	9 (69)	10 (100)	–	0.10	–
Bilateral abnormal MRI findings, n(%)	4 (31)	4 (40)	–	0.66	–
CSF pleocytosis (> 5µl), n (%)	3 (23)	1 (10)	–	0.43	–
Elevated CSF protein (> 40 mg/dl), n (%)	6 (46)	5 (50)	–	> 0.99	–
Elevated IgG index > 0.7, n (%)	1 (8)	0 (0)	–	> 0.99	–
Oligoclonal banding in CSF, n (%)	4 (31)	1 (10)	–	0.10	–
Abnormal EEG findings, n (%)	9 (69)	10 (100)	–	0.10	–
Tumor association, n (%)	3 (23)	2(20)	–	0.86	–

HC, healthy controls; NMDA, N-methyl-D-aspartate; MOG, myelin oligodendrocyte glycoprotein; LGI1, leucine-rich glioma-inactivated protein 1; CSF, cerebrospinal fluid, the two-sided unpaired t-test or Mann-Whitney U test were used for comparisons appropriately.

*Antibody-negative suspected autoimmune epilepsy, patients with antibodies negative for rat brain immunohistochemistry but met inclusion criteria and suspected autoimmune epilepsy.

### Proportion of plasmablasts was increased in patients with AE/Ab(+)

**Figure 1:**
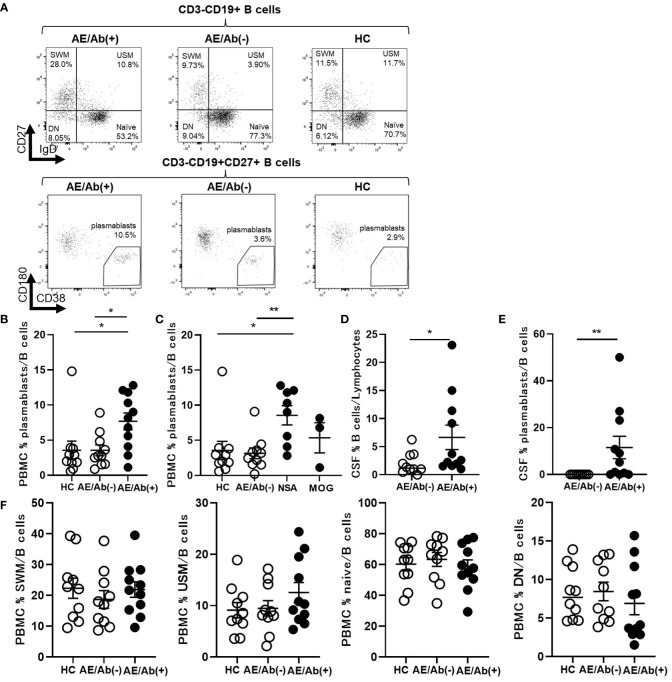
Prominent plasmablasts were found in patients with AE/Ab(+). **(A)** Representative flow cytometry analysis of CD19+CD27–IgD+ (naive), CD19+CD27–IgD– (DN), CD19+CD27+IgD+ (USM), CD19+CD27+IgD– (SWM), and CD19^int^CD27^high^CD38^high^CD180– (plasmablasts) in PBMCs of HC, patients with AE/Ab(-) and AE/Ab(+). **(B, C)** The frequency of plasmablasts in B cells of PBMCs of each group. NSA is defined as the patients group of NMDA receptor antibody-positive autoimmune epilepsy and LGI1 antibody associated autoimmune epilepsy. **(D)** The frequency of B cells within lymphocytes in the CSF of patients with AE/Ab(-) and AE/Ab(+). **(E)** The frequency of plasmablasts within B cells in the CSF of patients with AE/Ab(-) and AE/Ab(+). **(F)** The frequency of SWM, USM, naive, and DN within B cells in PBMCs of each group. HC (n=11), AE/Ab(-) (n=10), and AE/Ab(+) (n=11).Values are expressed as the mean ± SEM. *p < 0.05 and **p < 0.01; two-sided unpaired *t*-test or Mann-Whitney *U* test, as appropriate. HC, healthy controls; AE/Ab(-), antibody-negative suspected autoimmune epilepsy; AE/Ab(+), antibody-positive autoimmune epilepsy; SWM, switched memory B cell; USM, unswitched memory B cell; DN, double negative B cell; naive, naive B cell; NSA, neuronal surface antigen; NMDA, N-methyl-D-aspartate; LGI1, leucine-rich glioma-inactivated 1; MOG, myelin oligodendrocyte glycoprotein; PBMCs, peripheral blood mononuclear cells.

To elucidate the differences between the two groups, we analyzed the immune phenotype of B cells. The gating strategy for the B cell subsets is shown in **Supplementary Figure 1**. The frequency of plasmablasts in the B cells of PBMCs was higher in patients with AE/Ab(+) than in those with AE/Ab(-) and HC (**Figures 1A, B**). Next, we compared the AE/Ab(+) group positive for antibodies against the neuronal surface antigen (NSA) (NMDAR or LGI1 antibody-positive group) and MOG antibody-positive group (MOG). The frequency of plasmablasts in the B cells of PBMCs in patients with NSA was higher than that in patients with AE/Ab(-) and HC (**Figure 1C**). Furthermore, we found that the frequency of B cells and plasmablasts within B cells of the CSF was elevated in patients with AE/Ab(+) compared to that in patients with AE/Ab(-) (**Figures 1D, E**). There were no differences among patients with AE/Ab(+), AE/Ab(-), and HC with respect to the frequencies of naive B cells, USM, SWM, and DN in the PBMCs (**Figure 1F**).

### ICOS expressing cTfh and cTfh subsets shifted to cTfh17 were prominent in patients with AE/Ab(+)

**Figure 2:**
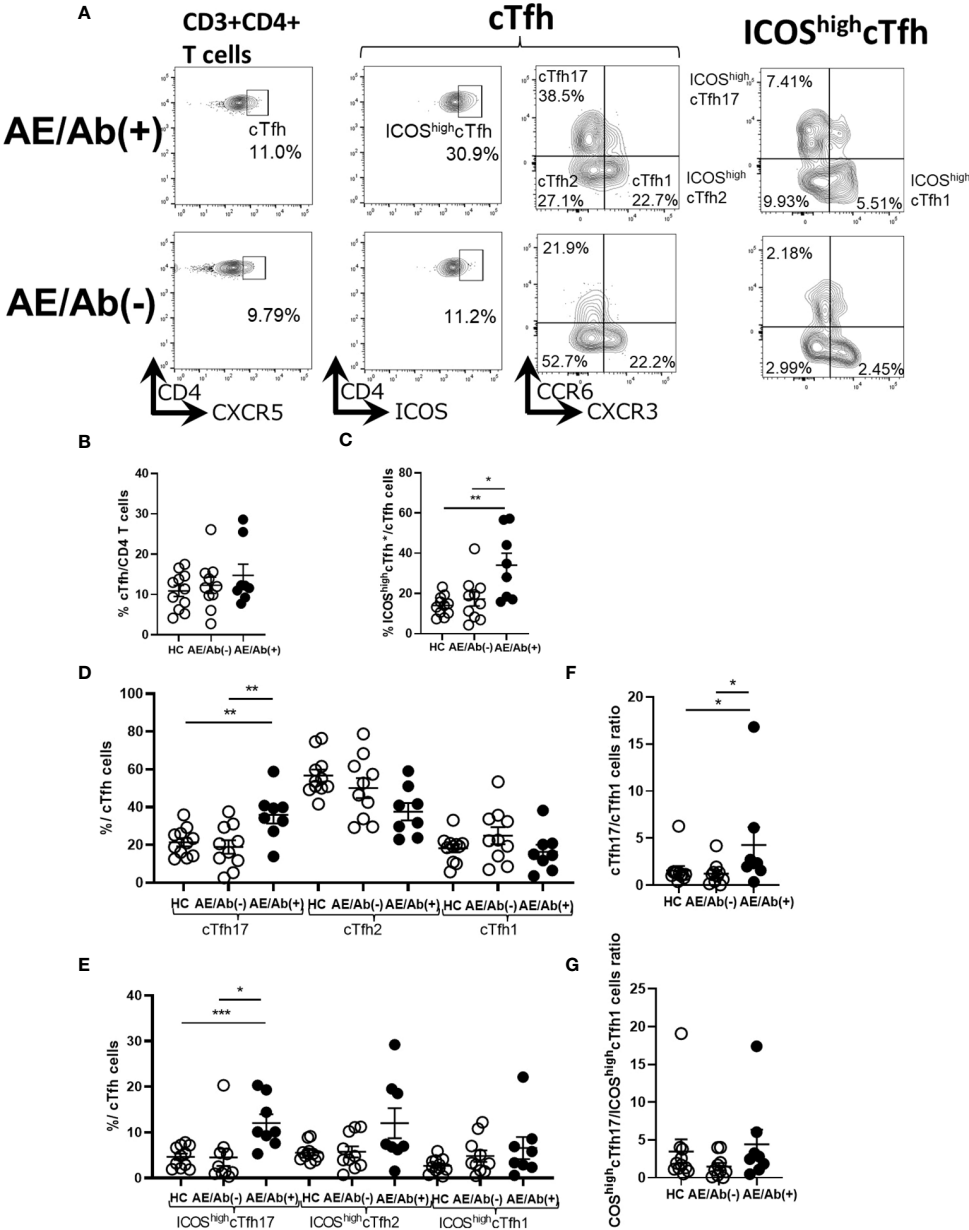
cTfh subset shifts to induce B cell differentiation in patients with AE/Ab(+). **(A)** Representative flow cytometry analysis of CD3+CD4+CXCR5+ T cells(cTfh), CD3+CD4+CXCR5+ICOS^high^CD4 T cells (ICOS^high^cTfh), CXCR3+CCR6−CXCR5+CD4 T cells(cTfh1), CXCR3−CCR6−CXCR5+CD4 T cells (cTfh2), CXCR3−CCR6+CXCR5+CD4 T cells (cTfh17), CXCR3+CCR6−CXCR5+ICOS^high^CD4 T cells (ICOS^high^cTfh1), CXCR3−CCR6−CXCR5+ICOS^high^CD4 T cells (ICOS^high^cTfh2), and CXCR3−CCR6+CXCR5+ICOS^high^CD4 T cells (ICOS^high^cTfh17) in PBMCs. **(B)** The frequency of cTfh within CD4 T cells in HC, patients with AE/Ab(-) and AE/Ab(+). **(C)** The frequency of ICOS^high^cTfh within cTfh in HC and patients with AE/Ab(-), AE/Ab(+). **(D)** The frequency of cTfh17, cTfh2, and cTfh1 within cTfh in HC, patients with AE/Ab(-) and AE/Ab(+). **(E)** The frequency of ICOS^high^cTfh17, ICOS^high^cTfh2, and ICOS^high^cTfh1 of cTfh in HC and patients with AE/Ab(-) and AE/Ab(+). **(F)** cTfh17/cTfh1 cells ratio of cTfh in HC, patients with AE/Ab(-) and AE/Ab(+). **(G)** ICOS^high^cTfh17/ICOS^high^cTfh1 cells ratio in HC, patients with AE/Ab(-) and AE/Ab(+). HC (n=11), AE/Ab(-) (n=10), and AE/Ab(+) (n=8). Values are expressed as the mean ± SEM. *p < 0.05, **p < 0.01 and ***p < 0.001; two-sided unpaired *t*-test or Mann-Whitney *U* test, as appropriate. HC, healthy controls; AE/Ab(-), antibody-negative suspected autoimmune epilepsy; AE/Ab(+), antibody-positive autoimmune epilepsy; cTfh, circulating follicular helper T cell; ICOS, inducible T-cell co-stimulator.

Since we found an increase in differentiating B cell subsets in patients with AE/Ab(+), we analyzed cTfh subsets and their ICOS expression in PBMCs (**Figure 2A**). The gating strategy for the cTfh subsets is shown in **Supplementary Figure 1**. The frequency of cTfh did not differ between patients with AE/Ab(+), AE/Ab(-) and HC (**Figure 2B**). In contrast, the frequency of ICOS^high^cTfh in cTfh was higher in patients with AE/Ab(+) than in those with AE/Ab(-) and HC (**Figure 2C**). In the subset analysis of cTfh, the frequency of cTfh17 in cTfh was higher in patients with AE/Ab(+) than in those with AE/Ab(-) and HC (**Figure 2D**), and the frequency of ICOS^high^cTfh17 in cTfh was particularly elevated in patients with AE/Ab(+) than in those with AE/Ab(-) and HC (**Figure 2E**). Furthermore, the cTfh17/cTfh1 cells ratio was higher in patients with AE/Ab(+) than in those with AE/Ab(-) and HC (**Figure 2F**). The ICOS^high^cTfh17/ICOS^high^cTfh1 cells ratio did not differ between each group (**Figure 2G**).

### The frequency of USM was associated with that of ICOS^high^cTfh17

**Figure 3:**
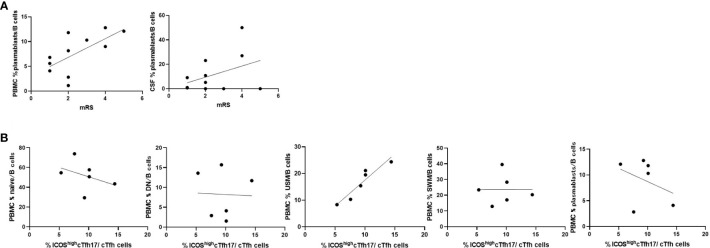
Frequencies of B cell subsets were associated with clinical severity and Tfh subsets in patients with AE/Ab(+). **(A)** Scatter plot of the frequency of plasmablasts within B cells in PBMCs or CSF and mRS on admission of patients with AE/Ab(+) (n = 11). The frequency of plasmablasts within B cells in PBMCs were associated with mRS (Spearman correlation coefficient, *r* = 0.67, *p* = 0.025). **(B)** Scatter plot of the frequency of B cell subsets within B cells in PBMCs and the frequency of ICOS^high^cTfh17 within cTfh cells in PBMCs of patients positive for antibodies against NSA (n = 6). The frequency of USM within B cells in PBMCs were associated with the frequency of ICOS^high^cTfh17 (Spearman correlation coefficient, *r* = 0.93, *p* = 0.0059). PBMCs, peripheral blood mononuclear cells; mRS, modified Rankin scale; cTfh, circulating follicular helper T cell; ICOS, inducible T-cell co-stimulator; naive, naive B cell; DN, double negative B cell; USM, unswitched memory B cell; SWM, switched memory B cell.

We then compared the immune phenotypes of AE/Ab(+) patients with their clinical manifestations. Our data showed an association between plasmablasts in B cells of PBMCs and the modified Rankin Scale (mRS) in patients with AE/Ab(+) on admission (**Figure 3A**). Furthermore, we analyzed whether the frequency of cTfh subsets was correlated with B cell subsets and found a positive correlation between USM in B cells of PBMCs and ICOS^high^cTfh17 in cTfh of patients with NSA (**Figure 3B**).

### Proportion of plasmablasts and ICOS^high^cTfh17 was increased in patients with NSA antibody positive autoimmune epilepsy

To assess the consistency of our results in each disease entity with NSA, we compared the phenotypes of B cell and cTfh subsets of patients with NSA. The frequency of plasmablasts in B cells of PBMCs was higher in patients with NMDA antibodies than in those with AE/Ab(-) and HC (**Supplemental Figure 2A**). Furthermore, the frequency of ICOS^high^cTfh17 in cTfh was higher in patients with NMDA and LGI1 antibodies than HC and was higher in patients with NMDA antibodies than patients with AE/Ab(-) (**Supplementary Figure 2B**).

### Proinflammatory cytokine levels were elevated in the serum and CSF of patients with AE/Ab(+)

**Figure 4:**
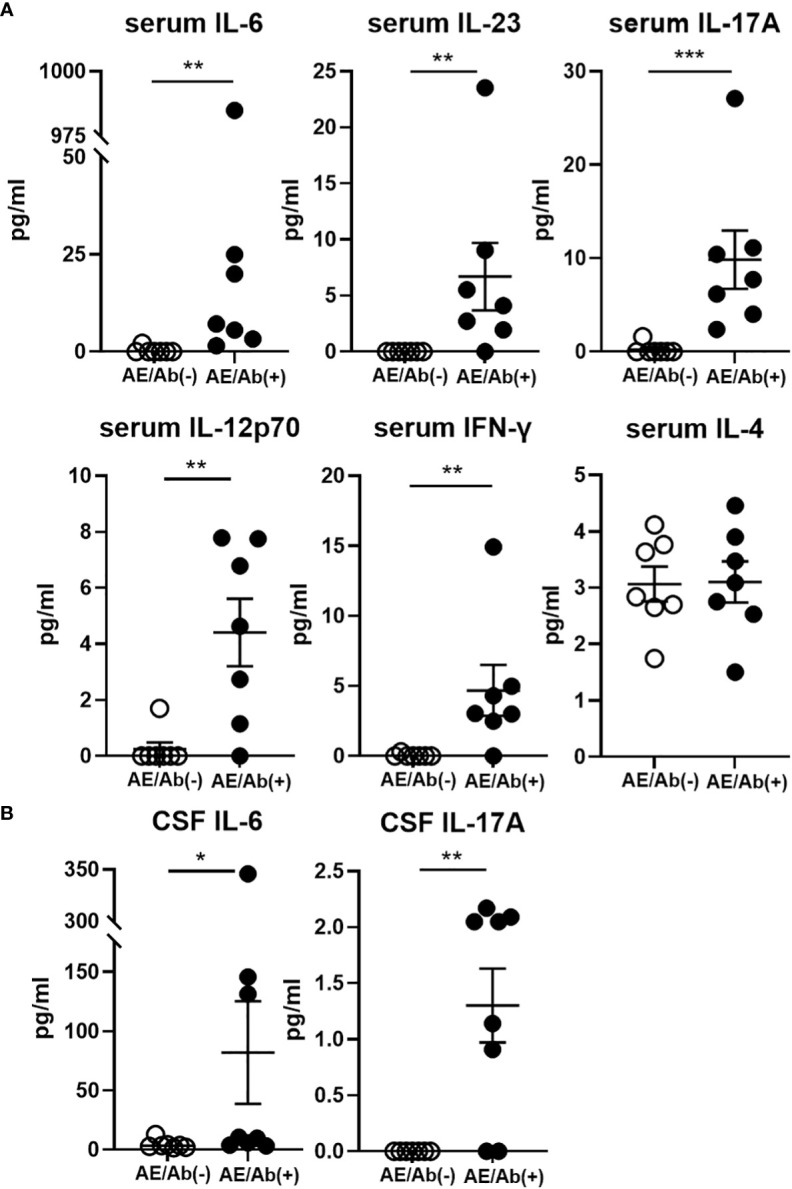
Serum and CSF levels of cytokines (pg/ml) in patients with AE/Ab(-) and AE/Ab(+). **(A)** Serum levels of cytokines (pg/ml) in patients with AE/Ab(-) (n=8) and AE/Ab(+) (n=7). **(B)** CSF levels of cytokines (pg/ml) in patients with AE/Ab(-) (n=8) and AE/Ab(+) (n=8). Values are expressed as the mean ± SEM. * p < 0.05, **p < 0.01, and *** p <0.001; two-sided unpaired *t*-test or Mann-Whitney *U* test, as appropriate. AE/Ab(-), antibody-negative suspected autoimmune epilepsy; AE/Ab(+), antibody-positive autoimmune epilepsy.

Finally, we measured the proinflammatory cytokine profile in the serum and CSF of patients with AE/Ab(+) and AE/Ab(-). Although the levels of cytokines were heterogeneous in the serum, IL-6, IL-23, IL-17A, IL-12p70, and IFN-γ levels were elevated in patients with AE/Ab(+) compared to those in patients with AE/Ab(-) (**Figure 4A**). In the CSF, IL-6 and IL-17A levels were elevated in patients with AE/Ab(+) compared to those with AE/Ab(-) (**Figure 4B**).

## Discussion

Currently, more than 20 anti-neuronal antibodies support the diagnosis of AE. Recent advances in these findings have facilitated recognition of AE cases. AE is a subtype of autoimmune encephalitis with seizure as a clinical symptom. Since anti-neuronal antibody tests are not always available, efforts have been made to diagnose AE without anti-neuronal antibodies testing based on clinical symptoms, imaging, and laboratory testing (5, 6); however, a more comprehensive analysis would be more accurate in preventing misdiagnosis of treatable diseases. Understanding multiple immune cell responses in disease pathogenesis could be a more appropriate strategy to improve the efficacy of AE diagnosis. In this study, we compared antibody-positive and antibody-negative patients with suspected autoimmune epilepsy. We did not observe any differences in clinical parameters, such as MRI findings, CSF markers, EEG findings, and age and sex, between the groups. In contrast, lymphocyte subset analysis of PBMCs showed that plasmablasts were elevated in the antibody-positive group, and the subset of cTfh cells was altered to stimulate B cell antibody production. In a previous study on antibody-positive and antibody-negative cases of autoimmune epilepsy or limbic encephalitis, antibody-positive cases were more likely to show MRI changes in the medial temporal lobes, and antibody-negative cases were more frequent in older men (8, 9). There were no differences in MRI changes, age, or sex between the antibody-positive and antibody-negative groups. These results highlight the difficulty in clinically diagnosing patients with autoimmune epilepsy.

We found that plasmablasts in PBMCs were elevated in patients with AE/Ab(+) compared to those in patients with AE/Ab(-) and HC. Elevated levels of plasmablasts are involved in the pathogenesis of some autoantibody-associated autoimmune diseases such as systemic lupus erythematosus (SLE), neuromyelitis optica spectrum disorder (NMOSD), and IgG4-related disorders (27–29). Especially in NMOSD, an autoantibody-associated autoimmune disease of the central nervous system (CNS), plasmablasts are elevated in PBMCs and CSF (27) and produce pathogenic autoantibodies (30, 31). Emerging evidence showing the dynamics of plasmablasts related to disease activity in autoantibody-associated autoimmune diseases highlights the potential of this B cell subset as a representative immune phenotype, which may be called “autoimmune plasmablastosis”. In a study on NMDAR encephalitis, plasmablasts were reported to be elevated in the active phase of the disease in PBMCs and decreased after rituximab administration (32). In another report involving four cases of NMDAR encephalitis, plasma cells in the CSF were elevated in the active phase of the disease and decreased after treatment (33). In line with these previous findings, in our study, patients with AE/Ab(+) showed elevated plasmablasts in PBMCs and CSF, and the former were associated with global physical disability (mRS) upon admission, which may highlight the increase in plasmablasts as an outcome of the immune response of patients with AE/Ab(+). Of note, one patient with AE/Ab(-) and one HC showed a relative increase in plasmablasts in PBMCs, which may reflect heterogeneity of the frequency of plasmablasts in peripheral circulation. Otherwise, other unknown autoantibodies may be present in patients with AE/Ab(-). Further investigation focusing on B cell phenotypes, regardless of anti-neural antibodies, would be worthwhile in future studies.

We further found that cTfh in patients with AE/Ab (+) strongly expressed ICOS. ICOS is preferentially expressed on Tfh; it is required for active regulation of B cell responses by interacting with ICOS ligands on B cells and is involved in IgG production (34, 35). Although ICOS^high^cTfh has been reported to be elevated in myasthenia gravis and idiopathic thrombocytopenic purpura (16, 36), elevated ICOS^high^cTfh levels in autoimmune epilepsy found in this study may reflect a common immunological feature of these diseases. Notably, cTfh was shifted to cTfh17 in the present study. cTfh17 is characterized by the production of the cytokine IL-17 and expression of the transcription factor ROR-γt, which has been shown to have a potent stimulatory effect on plasmablasts that produce IgG in autoimmune diseases, such as dermatomyositis (15). Several reports have shown that cTfh shifts to a subset of cTfh17 or cTfh2 in autoantibody-associated autoimmune diseases (16–19) that is consistent in patients with AE/Ab(+). Moreover, the cTfh17/cTfh1 cells ratio correlates with enhanced humoral immune response (20). In line with this, our data showed that cTfh shifted to cTfh17 in patients with AE/Ab(+), and the cTfh17/cTfh1 ratio increased. Indeed, ICOS^high^cTfh17 within cTfh were elevated in the patients with NMDA and LGI1 antibodies positive autoimmune epilepsy compared with AE/Ab(-) patients or HC. The ICOS^high^cTfh17/ICOS^high^cTfh1 ratio did not differ in each group, but It might be because ICOS is widely expressed among cTfh cell subsets in patients with AE/Ab(+).These results suggest that AE/Ab(+) shares a similar immune background with other autoantibody-associated autoimmune diseases.

In our study, the frequency of ICOS^high^cTfh17 within cTfh cells correlated with USM among the B cell subsets in patients with NSA. USMs are B-cell subsets that enter the germinal center and have the potential for early differentiation into plasmablasts supported by Tfh (37, 38). It has recently been reported that an increase in USM in the peripheral circulation is associated with IgG1/IgM responses to SARS-CoV2 results in earlier COVID-19 recovery (39). Furthermore, a parallel increase in circulating USM with cTfh has been reported in cancer patients who respond well to immune checkpoint therapy (40). Such an immune response in infection and cancer, in turn, potentially suggests an association between USM and cTfh in the autoimmune response in patients with NSA. Notably, this trend was not observed in patients with MOG antibodies, which requires further investigation for the immune background of their autoantibody production. In contrast, USM decreases during the active phase of established disease in SLE and rheumatoid arthritis and is negatively correlated with disease severity (41, 42). Although we did not find a decrease in USM but rather an increase in patients with AE/Ab(+), the differences may reflect that we enrolled patients in initial disease stages before the establishment of pathologically switched memory B cells.

Next we found IL-12p70, IL-23, IL-6, IL-17A, and IFN-γ levels were elevated in the serum, and IL-6 and IL-17A levels were elevated in the CSF of patients with AE/Ab(+) compared to those of patients with AE/Ab(-). Some studies have reported that cytokine changes in autoimmune encephalitis have focused on the CSF. Elevated levels of IL-6, IL-17A, and CXCL13, which is a ligand of CXCR5 and is a known B-cell-attracting chemokine in CSF, have been reported in NMDAR encephalitis (43, 44). Elevated IL-17A levels in the CSF have also been reported in non-NMDA autoimmune encephalitis (45), suggesting that CSF IL-17A is likely to play an important role in autoimmune encephalitis. In this study, we found elevated IL-6 and IL-17A levels in the CSF, similar to previous reports, and cytokine changes in the serum. IL-12 and IL-23, which are elevated in the serum, activate STAT4 and STAT3, respectively, and promote T-cell differentiation into the Tfh lineage (12, 46). IL-12 also induces Th1 differentiation and IFN-γ production in T cells (47). Elevated levels of IL-12 and IL-23 may induce subset changes in cTfh cells. IL-17 is a pro-inflammatory cytokine produced not only by Th17 cells, but also by Tfh17 (12). IL-17 downregulates tight junction molecules and facilitates leukocyte passage through the blood-brain barrier. IL-6 is also a pro-inflammatory cytokine that is elevated in CNS autoimmune diseases such as NMO, stimulates B cell differentiation (48), promotes plasmablasts survival, and enhances antibody secretion (30). Importantly, the levels of cytokines were less uniform in our AE/Ab(+) cases, which was probably due to the complex immune response in the active phase of AE. Our deeper immune phenotype analysis revealed clearer characteristic cTfh and B cell phenotypes relevant to changes in proinflammatory cytokines.

The shift in cTfh subsets, elevated plasmablasts, and cytokine changes that support their differentiation in peripheral blood suggest that the differentiation of B cells into plasmablasts in AE/Ab(+) takes place outside the CNS. In fact, among autoimmune encephalitis, NMDAR encephalitis does not form lymphatic follicles in brain pathology, but teratomas develop into tertiary lymphatic follicles, and teratoma-derived lymphocytes or the peripherally circulating B cells differentiate into plasmablasts and produce NR1 antibodies specific to NMDAR encephalitis (43, 49, 50). In LGI1 encephalitis, B cells in the CSF and plasmablasts of PBMCs have been reported to be clonally related (51). On the basis of these evidences, Dalmau et al. proposed the pathophysiology of autoimmune encephalitis. Naive B cells experience neuronal antigens in regional lymph nodes outside the CNS and then differentiate into memory B cells and antibody-producing cells *via* Tfh or other pathways. Memory B cells migrate to the CNS and differentiate into antibody-producing cells (52, 53). Our results support these hypotheses and further extend them as there is an alternative pathway for B cell differentiation in peripheral lymphoid organs.

This study had some limitations. First, it was a small-scale study. Although it is the same antibody-related autoimmune disease, the phenotype of each anti-neuronal antibody was difficult to compare due to limited number of available patients would require further evaluation in the future. Moreover, since this study was performed in cross-sectional way, we may need to evaluate patients in multiple points longitudinally to see future immune alteration that did not found in the initial evaluation. Second, the low number of cells in the CSF precluded the comparison of the subset analyses of cTfh in CSF. Third, we were unable to analyze patients with antibodies against intracellular antigens. Hence, a long-term prospective study is needed to determine the phenotype of each antibody in a large number of patients, and to consider the clinical manifestations of cTfh17, ICOS^high^cTfh17, and plasmablasts. Nevertheless, immune phenotype alterations with increased levels of proinflammatory cytokines in the serum highlighted the role of the autoimmune response in the peripheral lymphoid organs during the active phase of patients with AE/Ab(+).

## Conclusion

Given the changes in the subsets of B cells and cTfh cells that promote antibody production observed in the PBMCs of patients with antibody-positive autoimmune epilepsy, our data provide immune-related markers for representing antibody-positive autoimmune epilepsy. In particular, elevated plasmablasts levels and ICOS-expressing cTfh17 shift may provide a new diagnostic and therapeutic indicator for antibody-positive autoimmune epilepsy.

## Data availability statement

The original contributions presented in the study are included in the article/**Supplementary material**. Further inquiries can be directed to the corresponding authors.

## Ethics statement

The studies involving human participants were reviewed and approved by the ethics committee of Kobe University Hospital (Nos.1381 and B190152). The patients/participants provided their written informed consent to participate in this study.

## Author contributions

Study design and conceptualization: AH and NC. Major role in data acquisition: AH and NC. Analysis or interpretation of the data: AH, NC, RA, RN, AT, HY, MK, YO, YK, TK, FL, K-PW, and RM. Drafting the manuscript for intellectual content: AH and NC. supervision of the entire study: NC and RM. All authors contributed to the article and approved the submitted version.
