# Supplementary material for: Circulating plasmablasts and follicular helper T-cell subsets are associated with antibody-positive autoimmune epilepsy
Source: Front Immunol. 2022 Dec 8;13:1048428. doi: 10.3389/fimmu.2022.1048428 (PMC9773883; doi:10.3389/fimmu.2022.1048428)
Supplement: Supplementary file 1 [file DataSheet_1.docx]

Supplementary Material

| **Supplementary Table 1** \| List of antibodies and immunofluorescence. | | | |
| --- | --- | --- | --- |
| Tfh subsets | | | |
| Target | Conjugate | Clone | Company |
| CD3 | FITC | UCHT1 | BD |
| CD4 | PerCP/Cy5.5 | SK3 | BD |
| CXCR5 | PE | J252D4 | BioLegend |
| CCR6 | PE/Cy7 | G034E3 | BioLegend |
| ICOS | APC/Cy7 | C398.4A | BioLegend |
| CXCR3 | Brilliant Violet 650 | G025H7 | BioLegend |
| B cell subsets | | | |
| Target | Conjugate | Clone | Company |
| CD38 | PE/Cy7 | HIT2 | BD |
| IgD | PE-CF594 | IA6-2 | BD |
| CD27 | Brilliant Violet 421 | O323 | BioLegend |
| CD180 | PE | MHR73-11 | BioLegend |
| CD19 | APC/Cy7 | HIB19 | BioLegend |
| CD3 | FITC | UCHT1 | BD |
| Dead cells were excluded using Fixable Viability Dye eFluor 506 (Thermo Fisher Scientific, Waltham, MA, USA) | | | |

| **Supplementary Table 2** \| List of phenotype and naming of subset | |
| --- | --- |
| **B cell subset** | **Phenotype** |
| plasmablasts | CD19^int^CD27^high^CD38^high^CD180- B cells |
| naive B cell | CD19+CD27-IgD+ B cells |
| unswitched memory B cell: USM | CD19+CD27+IgD+ B cells |
| switched memory B cell: SWM | CD19+CD27+IgD- B cells |
| double negative B cell: DN | CD19+CD27-IgD- B cells |
| **circulating Tfh cell subset** | **Phenotype** |
| circulating follicular helper T cell: cTfh | CD3+CD4+CXCR5+ |
| circulating follicular helper T cell Th1-like cell: cTfh1 | CXCR3+CCR6-CXCR5+CD4 T cells |
| circulating follicular helper T cell Th2-like cell: cTfh2 | CXCR3-CCR6-CXCR5+CD4 T cells |
| circulating follicular helper T cell Th17-like cell: cTfh17 | CXCR3-CCR6+CXCR5+CD4 T cells |
| ICOS high expressing circulating follicular helper T cell: ICOS^high^cTfh | CD3+CD4+CXCR5+ICOS^high^CD4 T cells |
| ICOS high expressing circulating follicular helper T cell Th1-like cell: ICOS^high^cTfh1 | CXCR3+CCR6-CXCR5+ICOS^high^CD4 T cells |
| ICOS high expressing circulating follicular helper T cell Th2-like cell: ICOS^high^cTfh2 | CXCR3-CCR6-CXCR5+ICOS^high^CD4 T cells |
| ICOS high expressing circulating follicular helper T cell Th17-like cell: ICOS^high^cTfh17 | CXCR3-CCR6+CXCR5+ICOS^high^CD4 T cells |


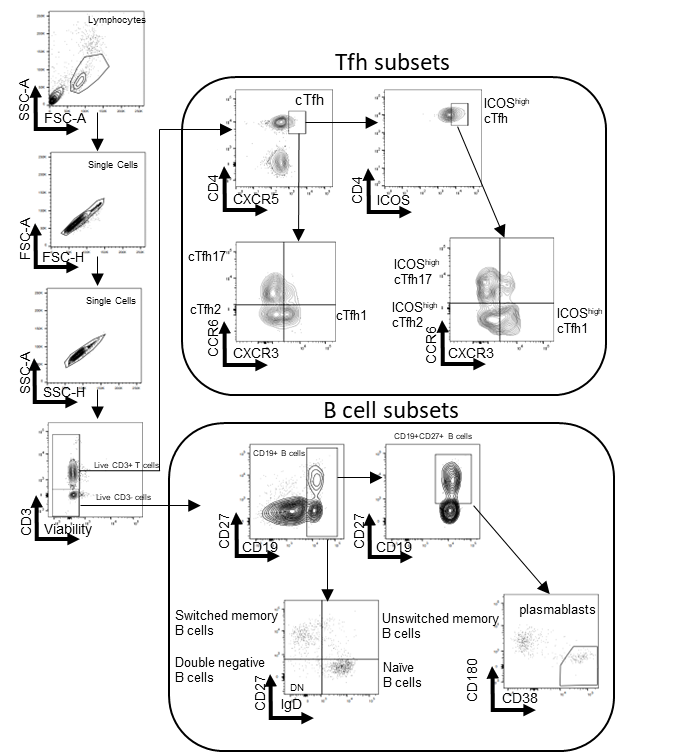


**Supplementary Figure 1. Gating strategy for cTfh and B cell subsets in PBMCs.**

After the removal of doublet from lymphocytes, live cells were divided into CD3+ T cells and CD3- non-T cells. CD3+ T cells were gated out for CXCR5+CD4+ (cTfh) or CXCR5+ICOS^high^CD4+ (ICOS^high^cTfh); then, based on the expressions of CXCR3 and CCR6, we annotated cTfh subsets as follows; CXCR3+CCR6–CXCR5+CD4+ (cTfh1), CXCR3–CCR6–CXCR5+CD4+ (cTfh2), and CXCR3–CCR6+CXCR5+CD4+ (cTfh17), CXCR3+CCR6–CXCR5+ICOS^high^CD4+ (ICOS^high^cTfh1), CXCR3–CCR6–CXCR5+ICOS^high^CD4+ (ICOS^high^cTfh2), and CXCR3–CCR6+CXCR5+ICOS^high^CD4+ (ICOS^high^cTfh17). In non-T cells, CD19+ B cells were gated out; then, based on the expressions of CD27 and IgD, we annotated CD19+ B cell subsets as follows; CD19+CD27–IgD+ (naïve B cell), CD19+CD27–IgD– (double negative B cell), CD19+CD27+IgD+ (unswitched memory B cell), and CD19+CD27+IgD– (switched memory B cell). We further gated out CD19+CD27+ cells and annotated CD19^int^CD27^high^CD38^high^CD180– as plasmablasts. PBMCs, peripheral blood mononuclear cells; cTfh, circulating follicular helper T cell; ICOS, inducible T-cell co-stimulator.

A　　　　　　　　　　　　B

**Supplementary Figure 2. Plasmablasts and ICOS^high^cTfh17 population in patients with NMDA and LGI1 antibody positive autoimmune epilepsy.**

(A) The frequency of CD19^int^CD27^high^CD38^high^CD180– (plasmablasts) in PBMCs of HC, patients with AE/Ab(-), NMDA and LGI1.(B)The frequency of ICOS^high^CXCR3–CCR6+CXCR5+CD4 T cells (ICOS^high^cTfh17) in PBMCs of HC, patients with AE/Ab(-), NMDA and LGI1. *p < 0.05, **p < 0.01, and *** p <0.001; two-sided unpaired *t*-test or Mann-Whitney *U* test, as appropriate. HC, healthy controls; AE/Ab(-), antibody-negative suspected autoimmune epilepsy; NMDA, N-methyl-D-aspartate receptor antibody positive autoimmune epilepsy; LGI1, leucine-rich glioma-inactivated 1 antibody positive autoimmune epilepsy; cTfh, circulating follicular helper T cell; ICOS, inducible T-cell co-stimulator.
